# Prevalence and Anatomic Distribution of Serrated and Adenomatous Lesions in Patients with Inflammatory Bowel Disease

**DOI:** 10.1155/2017/5490803

**Published:** 2017-01-15

**Authors:** Lik Hang Lee, Marietta Iacucci, Miriam Fort Gasia, Subrata Ghosh, Remo Panaccione, Stefan Urbanski

**Affiliations:** ^1^Department of Pathology and Laboratory Medicine, University of Calgary and Foothills Medical Centre, Calgary, AB, Canada; ^2^Department of Medicine, University of Calgary and Foothills Medical Centre, Calgary, AB, Canada

## Abstract

*Background*. Sessile serrated adenomas/polyps (SSA/Ps) and traditional serrated adenomas (TSAs) have not been well characterized in patients with inflammatory bowel disease (IBD). This study assesses the prevalence and anatomic distribution of SSA/Ps, TSAs, and conventional adenomas/dysplasia (Ad/Ds) in IBD patients.* Methods*. IBD patients with serrated, adenomatous, or hyperplastic lesions between 2005 and 2009 were identified in the regional tertiary-care hospital database. Clinicopathological information was reviewed and the histology of biopsies was reevaluated.* Results*. Ninety-six Ad/Ds, 25 SSA/Ps, and 4 TSAs were identified in 83 patients. Compared to Ad/Ds, serrated lesions were more prevalent in females (*p* = 0.046). The prevalence of Ad/Ds was 4.95%, SSA/Ps was 1.39%, and TSAs was 0.31%. No relationship was identified between lesion type and IBD type. Comparing all IBD patients, the distribution of lesion types was significantly different (*p* = 0.02) with Ad/Ds more common distally, SSA/Ps more common proximally, and TSAs evenly distributed. Among Crohn's disease (CD) patients, a similar distribution difference was noted (*p* < 0.001). However, ulcerative colitis (UC) patients had a uniform distribution of lesion types (*p* = 0.320).* Conclusions*. IBD patients have a lower prevalence of premalignant lesions compared to the general population, and the anatomic distribution of lesions differed between CD and UC patients. These findings may indicate an interaction between lesion and IBD pathogenesis with potential clinical implications.

## 1. Introduction

**Figure 1:**
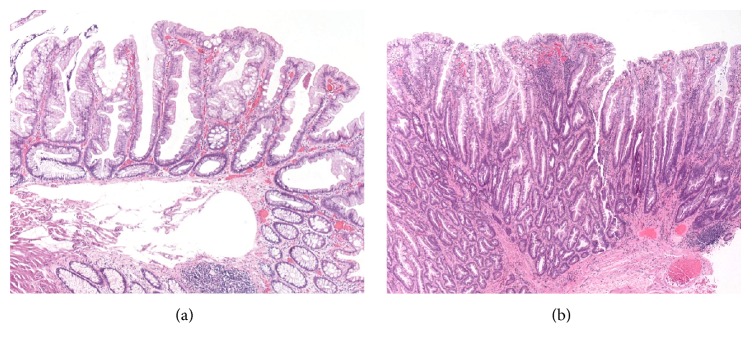
Representative histology with H&E staining of (a) a sessile serrated adenoma or polyp (SSA/P) and (b) a traditional serrated adenoma (TSA). SSA/Ps generally show no or only focal conventional cytological dysplasia, have basal flattening or dilation of crypts, and have exaggerated apical cytoplasm creating a serrated morphology. TSAs have cytologic dysplasia throughout, have hypereosinophilic cytoplasm, and have a serrated morphology formed primarily by multiple adjacent complex buds (ectopic crypts).

Since the time of the description of the serrated colonic lesions (sessile serrated adenomas or polyps (SSA/Ps) and traditional serrated adenomas (TSAs, Figure 1)), it has been known that these lesions will be encountered in the setting of the inflammatory bowel disease (IBD) [1, 2]. Their prevalence, however, in the setting of the IBD was far from certain given the fact that their detection rate was not known in the general population nor in the IBD patient population.

Since that time, significant progress has been made and some information is available regarding the distribution of these serrated lesions in the general population [3–6]. There are also communications addressing the issues of the serrated lesions in the IBD setting [7]. Considering the significant gaps in our understanding of many aspects of the serrated lesions arising in the background of the IBD, we have attempted to improve our understanding of these lesions by studying the population of IBD patients seen in our institution.

The aim of our study is to determine the prevalence and the anatomic distribution of SSA/Ps and TSAs in patients with IBD and to provide a comparison with conventional colonic adenomas/dysplasia (Ad/Ds) in the same population.

## 2. Materials and Methods

### 2.1. Patients

Patients were identified through a search of the pathology database of our metropolitan area (Calgary Laboratory Services) for all patients with colorectal involvement by IBD who have had a colorectal biopsy at the region's principal tertiary-care hospital during the years 2005–2009. Briefly, this involved searching for all terms related to IBD such as “colitis”, “Crohns”, and “inflammatory bowel disease”. Clinical charts of these patients were reviewed to identify patients with a final IBD diagnosis. Among patients with a diagnosis of IBD, those with colorectal involvement by pathologic or clinical assessment were selected and the pathology records for these patients were reviewed to identify those with the diagnosis of a serrated lesion, adenomatous lesion, or hyperplastic polyp (HP) on biopsy. The patients with at least one of these lesions were selected for histologic review. The structure of Alberta's provincial health care system and its medical health records permits an accurate capture and search of data.

### 2.2. Data Collection

The histology of gastrointestinal biopsies for the patients with slides that were available for review was rediagnosed by a gastrointestinal pathologist who was blinded to the original diagnoses. In cases of diagnostic difficulty, opinions from additional pathologists were requested. Patients may have had more than one endoscopic procedure. Premalignant lesions were identified and classified as Ad/Ds, SSA/Ps, and TSAs. Dysplasia-associated lesion or mass (DALM) and sporadic adenomas were not differentiated. All other lesions on rediagnosis (HPs, carcinoma, no pathologic abnormalities, etc.) were not analyzed further in this study. A chart review was performed to record detailed demographic and clinical factors such as patient gender, age, IBD type (Crohn's disease (CD) or ulcerative colitis (UC)), biopsy location, and lesion size.

### 2.3. Statistical Analysis

Baseline characteristics were analyzed with standard descriptive statistics and compared between the three types of premalignant lesions. Continuous variables were analyzed using one-way analysis of variance (ANOVA) or Student's *t*-test, depending on the number of groups. Categorical variables were compared using *χ* ^2^ or the Fisher exact test depending on the number of observations. A *p* value < 0.05 was considered statistically significant. Period prevalence of each lesion type was determined on a per-patient basis. It was defined as the cumulative identification of one or more of the lesion types in each patient over the time frame of the study. Analyses were performed using SPSS 19.0 for Windows (IBM Corp., Armonk, NY).

### 2.4. Ethical Statement

This study was approved by the ethics committee of the University of Calgary.

## 3. Results

There were 1484 unique patients with colorectal inflammatory bowel disease that had biopsies performed. Among these patients, there were 319 biopsies in 188 patients (12.7%) that were diagnosed as a serrated lesion, adenomatous lesion, or HP. Two hundred and eighty-eight of these biopsies in 164 patients were available for review.

After pathology review and rediagnosis, there were 96 Ad/Ds, 25 SSA/Ps, and 4 TSAs identified in 83 patients. Fifty-three lesions were in patients with CD, 66 in patients with UC, and 6 in patients with unclassified colitis. These were identified in 97 endoscopic procedures (including 8 sigmoidoscopies), of which 21 (21.6%) procedures identified two or more lesions of interest. Within our study period, 72 of the 83 patients only had a lesion of interest identified in one procedure, with prior and subsequent procedures not identifying an Ad/D, SSA/P, or TSA. Nine patients had lesions discovered in more than one procedure within the same year. Two patients had lesions discovered in more than one procedure over several years. The eight sigmoidoscopies were performed on seven patients. Subsequent colonoscopies within the study time period identified additional lesion of interest in two patients, while no additional lesions were identified in the other five patients.

Table 1 shows a summary of the demographics and characteristics of each lesion. The* p* value listed in the table is for the comparison between the characteristics of Ad/Ds and SSA/Ps. TSAs are excluded from this comparison due to small numbers. Sixty-four patients had one or more Ad/Ds, 18 patients had one or more SSA/Ps, and 4 patients had one or more TSAs. Of the 25 SSA/P lesions identified in our study, 15 (60.0%) were initially diagnosed as hyperplastic polyps, with only six originally signed out as an SSA/P. The remainder had various other diagnoses or were descriptive, such as “serrated lesion.”

The prevalence of each lesion type, on a per-patient basis, in the total IBD population is estimated. Because only 164 of the 188 patients (87.2%) had material available for review, we extrapolate for calculation that our total IBD study population is approximately 87.2% of the 1484 identified IBD patients or 1294 patients. Thus, the prevalence of each lesion is 4.95% for Ad/Ds (64 of 1294 patients), 1.39% for SSA/Ps (18 of 1294 patients), and 0.31% for TSAs (4 of 1294 patients). Of the 1484 total patients, 760 (51%) are male and 724 (49%) are female. By a similar method of extrapolation, the prevalence of Ad/Ds is therefore 5.43% in males compared to 4.43% in females, that of SSA/Ps is 1.06% in males compared to 1.74% in females, and that of TSAs is 0.15% in males compared to 0.48% in females.

**Figure 2:**
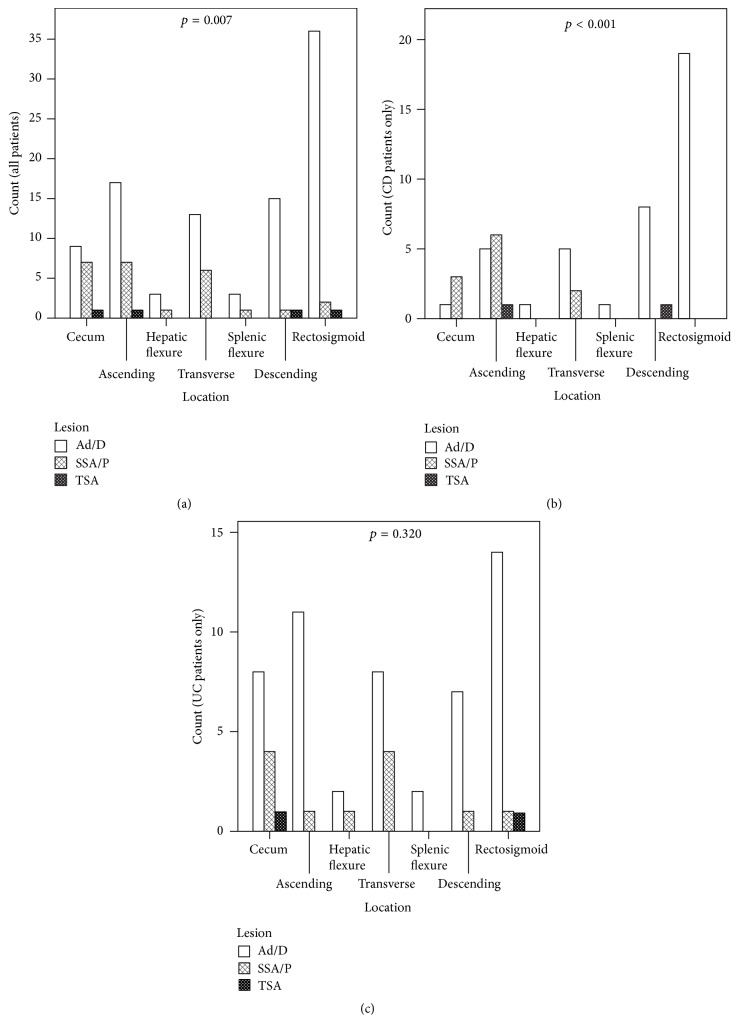
Anatomical distribution of conventional adenomas/dysplasia (Ad/Ds), sessile serrated adenoma or polyps (SSA/Ps), and traditional serrated adenomas (TSAs) for (a) all patients studied, (b) patients with Crohn's disease, and (c) patients with ulcerative colitis. *p* values are for the comparison between Ad/Ds and SSA/Ps.

**Figure 3:**
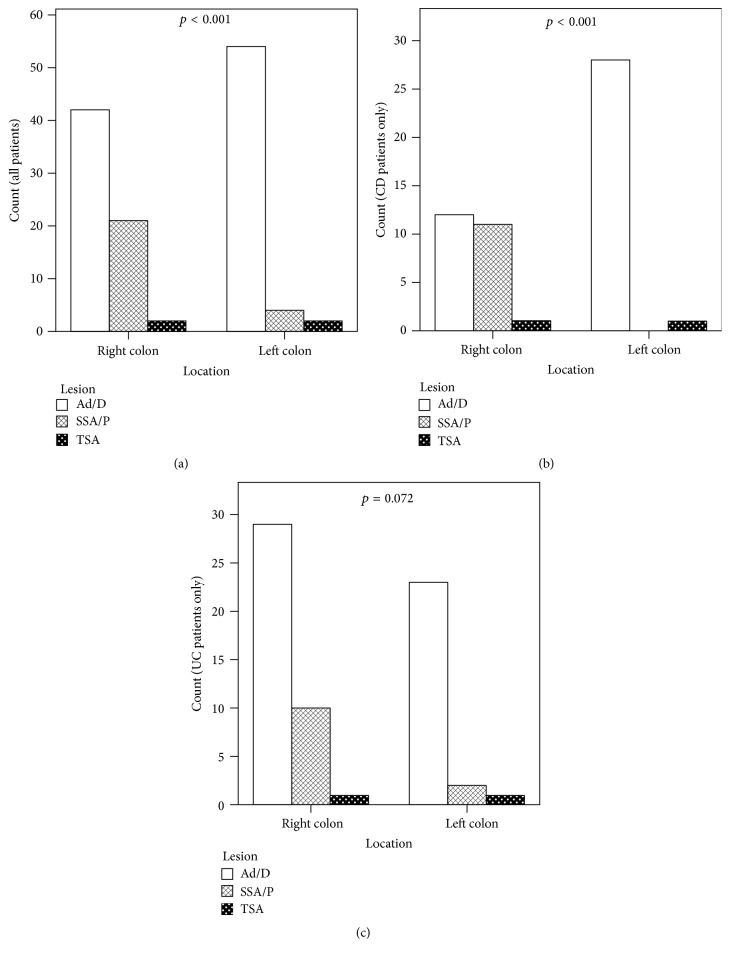
Anatomical distribution of conventional adenomas/dysplasia (Ad/Ds), sessile serrated adenoma or polyps (SSA/Ps), and traditional serrated adenomas (TSAs) grouped into right and left colon for (a) all patients studied, (b) patients with Crohn's disease, and (c) patients with ulcerative colitis. The right colon is defined as proximal to the splenic flexure, and the left colon is defined as distal to and including the splenic flexure. *p* values are for the comparison between Ad/Ds and SSA/Ps.

The distribution of diagnoses by anatomic location is listed in Table 1 and presented in Figure 2, with *p* values shown for comparison between Ad/Ds and SSA/Ps. The distribution grouped into right and left colon is shown in Figure 3. Right colon was defined as cecum, ascending colon, hepatic flexure, and transverse colon. Left colon was defined as splenic flexure, descending colon, and rectosigmoid colon. There is a difference in distribution between Ad/Ds and SSA/Ps among all patients, with a higher proportion of SSA/Ps in the right colon compared to the left, and a higher proportion of Ad/Ds in the left colon compared to the right. A similar distribution is noted when assessing only the CD patients. However, when assessing only the UC patients, there is no difference in distribution between Ad/Ds and SSA/Ps; There is a higher proportion of lesions in the right colon for both Ad/Ds and SSA/Ps. When looking at the breakdown of the distribution (Figure 2(c)), a more uniform distribution of lesions across the entire colon is identified.

A closer look at the patients reveals that 26 patients had multiple lesions. Seventeen patients had multiple Ad/Ds, some identified concurrently and some identified in multiple colonoscopies over time. Of these 17 patients, one had six Ad/Ds identified in three colonoscopies over three years with locations ranging from the ascending colon to the rectosigmoid. Six patients had two SSA/Ps; in five of these patients, their SSA/Ps were identified at the same colonoscopic procedure. Interestingly, of these six patients, one patient had SSA/Ps in widely separated anatomic locations identified in one procedure (one at the splenic flexure and one in the rectosigmoid), while the other five patients had their SSA/Ps identified in the same or adjacent anatomic locations.

Three patients had diagnoses of different lesions. One patient had six Ad/Ds and one SSA/P, all identified in one procedure. The Ad/Ds were located from the cecum to the rectosigmoid. The SSA/P was located in the cecum. The second patient had one Ad/D and two SSA/Ps identified in one procedure. All lesions were located in the ascending colon. The third patient had three Ad/Ds and one TSA diagnosed in two procedures four months apart. The Ad/Ds were all in the rectosigmoid, while the TSA was in the cecum.

## 4. Discussion

The management of IBD patients with premalignant lesions—Ad/Ds, SSA/Ps, and TSAs—is controversial due to the paucity of studies characterizing these lesions in this patient population. Our study is a large retrospective cohort study identifying the frequency and distribution of Ad/Ds, SSA/Ps, and TSAs in IBD patients and among the first study to closely examine the anatomic distribution of these polyps.

As expected from studies on the general non-IBD population, in our study, Ad/Ds were more often found in males than in females. The reverse was true for serrated lesions, consistent with the literature [3]; females had right-sided dominance of adenomas [8]. Similarly, a greater percentage of colorectal carcinomas in females were nonpolypoid [9]. Considering that nonpolypoid colorectal cancers would more likely arise from sessile polyps, including SSA/Ps, it is important to note that our findings suggest that this gender preference may continue to hold true in IBD patients.

Compared to the prevalence of premalignant lesions in the non-IBD population, our study found that the prevalence of these lesions in IBD patients was low at 4.95% for Ad/Ds, 1.39% for SSA/Ps, and 0.31% for TSAs. This is lower than the prevalence of conventional adenomas in the general population [10]. In fact, in 2006, the US Multisociety Task Force recommended, as a colonoscopy quality indicator, that adenomas be detected in at least 25% of men and 15% of women aged 50 and above, regardless of indication [11]. It is debatable whether these guidelines are applicable to any specific gastroenterology practice. Studies have found varying detection rates in different populations [4, 8, 12–14], and surveillance colonoscopies have higher rates than screening colonoscopies (37% versus 25%, resp., in Anderson et al.) [15]. In regard to SSA/Ps, studies have found a prevalence ranging from 2 to 7% in patients at an average risk for colorectal cancer [3–6] and of 14% in patients with a personal or family history of SSA/Ps [16]. TSAs are more rare, with one study detecting it in 0.6% of average-risk patients [4]. The lower prevalence of lesions in IBD patients found in our study is similar to that found in the few studies that have also examined the IBD population. Dixon et al. found three adenomatous polyps in a cohort of 106 IBD patients (2.8%) compared to 67 in 749 non-IBD control patients (8.9%) [17]. A study of the patients in Olmsted County, Minnesota, found 29 of 692 IBD patients (4.2%) with dysplasia after an average of 14-year follow-up [18].

The lower rates of lesions in IBD patients may be a result of methodology, biology, or both. Methodologically, the retrospective nature of our study may result in underestimation. Furthermore, IBD patients undergo colonoscopies at a relatively young age; the average age in our study is 56 years. The detection of adenomas is known to be significantly lower in the <50-year-old population [8]. It is also notable that inflammatory changes may mask lesions. This is evidenced by increased detection of dysplastic lesions in IBD patients with the use of improved endoscopic technology, such as high definition colonoscopy [19] and virtual chromoendoscopy [20]. Biologically, IBD is a known risk factor for colorectal cancer [21]. Whether this is biologically intrinsic or a result of poor detection of dysplastic lesions in the inflamed colon is unknown. However, there is suggestion that IBD treatments, such as 5-ASA or immune modulation, may be protective against adenomas [22–24]. Furthermore, alternative mechanisms to the traditional adenoma-carcinoma sequence, such as the serrated pathway, may be important in colorectal carcinomas in IBD patients. Investigation of the serrated pathway and serrated lesions in IBD patients is nascent. Only one previous study looked at the prevalence of SSA/Ps in IBD patients, finding 9 cases in 4208 patients (0.21%) in a 3-year time frame [25]. Nevertheless, because the prevalence of SSA/Ps in IBD patients is much lower than Ad/Ds, the contribution of the serrated pathway to carcinoma likely remains comparatively small.

Of particular interest is the anatomic distribution of the lesions in IBD patients (Figure 2). In our IBD population, Ad/Ds were much more common in the rectosigmoid than in other regions of the colon and are overall more often left-sided. SSA/Ps, however, were more often right-sided. This is similar to the distribution found in the general population [13, 26–29]. Similar findings were seen when examining the CD population. This difference in distribution was statistically significant. However, the UC population had a different anatomic distribution of these lesions; both Ad/Ds and SSA/Ps were more evenly distributed throughout the colon. In the UC population, when anatomic locations are grouped into right and left sides (Figure 3), both Ad/Ds and SSA/Ps were more prevalent in the right colon. The difference in distribution between Ad/Ds and SSA/Ps in UC was not statistically significant. This is an interesting observation that has not been previously reported. The reason for such a distribution is unknown and can only be speculated. Perhaps it resulted from the more distal involvement of inflammation in UC patients masking lesions, or resulted from adenomatous lesions being less prevalent in inflamed background tissue. We were not always able to determine whether lesions came from a portion of the colon involved or not involved by colitis on either endoscopy or histology, making this confounding factor difficult to assess. The pathogenesis of the lesions may also interact with differences in the pathogenesis between CD and UC, including environmental risk factors and genetics [30–32]. As there has not been a previous study on the distribution of Ad/Ds and SSA/Ps in IBD patients, confirmation of our observation requires further investigation.

Our study also looked at the distribution of TSAs in IBD patients, which was a rare finding. The four TSAs identified were equally distributed with two in the left and two in the right colon. Furthermore, two were in UC and two were in CD patients, again, each with one on each side of the colon. Of note, the rate of TSAs in our population was lower than Ad/Ds or SSA/Ps by a proportion similar to that in the general population. However, the small numbers of TSAs in our study made our findings inconclusive. Furthermore, our findings differ from the left-sided predominance of TSAs in the general population [29, 33].

There are limitations to our study. First, IBD patients were identified through a database search. Although cases flagged by the search were corroborated with a chart review, some patients may be missed. Furthermore, as this was a retrospective study, an underestimation of premalignant lesions may occur, particularly for lesions that are more subtle such as SSA/Ps. Our study includes all biopsies, including both colonoscopies and sigmoidoscopies. As a result, the left colon may have been assessed more often, increasing the number of left-sided lesions. However, this bias would affect CD and UC patients equally and thus would not change the finding that CD and UC patients have different lesion distributions. More importantly, sigmoidoscopies were followed by subsequent colonoscopies. Another limitation is that we determined prevalence over a five-year time frame and included multiple endoscopy procedures if performed within this time frame. This may bias the results as new lesions may develop over time and some patients may have had closer endoscopic follow-up than others. However, only two patients had lesions discovered on more than one procedure that were performed a year or more apart. Finally, we were often unable to assess whether the lesions were found in a region of colitis and did not consider variables such as the clinical subtypes of UC and CD. Ongoing prospective studies are being performed to address these issues.

In conclusion, our study found that IBD patients had a lower prevalence of all types of premalignant lesions compared to published data on the general population. Ad/Ds remain the most common, followed by SSA/Ps. TSAs were the least common. The proportion of each lesion type was not different than the general population. As the first study on the distribution of these lesions in IBD patients, we found a left-sided prevalence of Ad/Ds and a right-sided prevalence of SSA/Ps, similar to the general population, in the total IBD population as well as in the population of CD patients. However, a more uniform distribution was identified in the UC population, which is an important finding that warrants further clinical and pathobiological investigation.

## Figures and Tables

**Table 1:** Patient and premalignant lesion characteristics.

	Ad/D	SSA/P	TSA	All lesions	*p* value^a^
*Assessment by patient* ^b^ (*N* (% of patients with each lesion))					

Number of patients (% of all patients with lesions of interest)	64 (77.1)	18 (21.7)	4 (4.8)	83	<0.001
Gender					0.193
M	36 (56.3)	7 (38.9)	1 (25.0)	42 (50.6)	
F	28 (43.8)	11 (61.1)	3 (75.0)	41 (49.4)	
Age (years, mean ± SD)	56.4 ± 14.3	55.3 ± 13.4	56.3 ± 19.1	56.0 ± 14.2	0.762
IBD type					1.000
UC	30 (46.9)	8 (44.4)	2 (50.0)	38 (45.8)	
CD	30 (46.9)	9 (50.0)	2 (50.0)	40 (48.2)	
Unclassified	4 (6.3)	1 (5.6)	0 (0.0)	5 (6.0)	

*Assessment by lesion* (*N* (% of lesion type))					

Number of lesions (% of all lesions of interest)	96 (76.8)	25 (20.0)	4 (3.2)	125	<0.001
Gender					0.046
M	56 (58.3)	9 (36.0)	1 (25.0)	66 (52.8)	
F	40 (41.7)	16 (64.0)	3 (75.0)	59 (47.2)	
Age (years, mean ± SD)	56.5 ± 13.8	55.3 ± 13.4	53.3 ± 20.0	56.2 ± 13.8	0.702
Size^c^ (cm, mean ± SD)	0.58 ± 0.39	0.61 ± 0.34	0.73 ± 0.25	0.59 ± 0.38	0.711
IBD type					0.696
UC	52 (54.2)	12 (48.0)	2 (50.0)	66 (52.8)	
CD	40 (41.7)	11 (44.0)	2 (50.0)	53 (42.4)	
Unclassified	4 (4.2)	2 (8.0)	0 (0.0)	6 (4.8)	
Location					
Cecum	9 (9.4)	7 (28.0)	1 (25.0)	17 (13.6)	
Ascending colon	17 (17.7)	7 (28.0)	1 (25.0)	25 (20.0)	
Hepatic flexure	3 (3.1)	1 (4.0)	0 (0.0)	4 (3.2)	
Transverse colon	13 (13.5)	6 (24.0)	0 (0.0)	19 (15.2)	
Splenic flexure	3 (3.1)	1 (4.0)	0 (0.0)	4 (3.2)	
Descending colon	15 (15.6)	1 (4.0)	1 (25.0)	17 (13.6)	
Rectosigmoid	36 (37.5)	2 (8.0)	1 (25.0)	39 (31.2)	
Location					
Right colon	42 (43.8)	21 (84.0)	2 (50.0)	65 (52.0)	
Left colon	54 (56.3)	4 (16.0)	2 (50.0)	60 (48.0)	

^a^Comparison between Ad/Ds and SSA/Ps only.

^b^Some patients have more than one lesion type.

^c^If there are multiple fragments, it represents size of largest fragment.

Ad/D, conventional adenomas/dysplasia; CD, Crohn's disease; IBD, inflammatory bowel disease; SSA/P, sessile serrated adenoma or polyp; SD, standard deviation; TSA, traditional serrated adenoma; UC, ulcerative colitis.
